# Phosphorylcholine Allows for Evasion of Bactericidal Antibody by *Haemophilus influenzae*

**DOI:** 10.1371/journal.ppat.1002521

**Published:** 2012-03-01

**Authors:** Sarah E. Clark, Julian Snow, Jianjun Li, Tracey A. Zola, Jeffrey N. Weiser

**Affiliations:** 1 Department of Microbiology, University of Pennsylvania, Philadelphia, Pennsylvania, United States of America; 2 Department of Chemistry and Biochemistry, University of the Sciences, Philadelphia, Pennsylvania, United States of America; 3 Institute for Biological Sciences, National Research Council Canada, Ottawa, Ontario, Canada; Fred Hutchinson Cancer Research Center, United States of America

## Abstract

The human pathogen *Haemophilus influenzae* has the ability to quickly adapt to different host environments through phase variation of multiple structures on its lipooligosaccharide (LPS), including phosphorylcholine (ChoP). During colonization with *H. influenzae*, there is a selection for ChoP+ phase variants. In a murine model of nasopharyngeal colonization, this selection is lost in the absence of adaptive immunity. Based on previous data highlighting the importance of natural antibody in limiting *H. influenzae* colonization, the effect of ChoP expression on antibody binding and its bactericidal activity was investigated. Flow cytometric analysis revealed that ChoP+ phase variants had decreased binding of antibody to LPS epitopes compared to ChoP− phase variants. This difference in antibody binding correlated with increased survival of ChoP+ phase variants in the presence of antibody-dependent, complement-mediated killing. ChoP+ phase variants were also more resistant to trypsin digestion, suggesting a general effect on the physical properties of the outer membrane. Moreover, ChoP-mediated protection against antibody binding correlated with increased resilience of outer membrane integrity. Collectively, these data suggest that ChoP expression provides a selective advantage during colonization through ChoP-mediated effects on the accessibility of bactericidal antibody to the cell surface.

## Author Summary

The bacterial pathogen *Haemophilus influenzae* evades immune responses during colonization in its human host. Decoration of the bacterial surface with different structures is one way that *Haemophilus* avoids host recognition. In this study, we show that the attachment of the small molecule phosphorylcholine, or ChoP, to the lipopolysaccharide covering the bacterial surface allows *H. influenzae* to avoid the immune response by inhibiting antibody binding. The presence of ChoP alters the bacterial surface to reduce its accessibility. The ability of ChoP to affect antibody binding is dependent on the positive charge of the molecule, which changes the physical properties of the bacterial membrane. The increased survival of bacteria with ChoP attached to their surface enriches ChoP+ bacteria during colonization. This study reveals a novel mechanism for bacterial evasion of a host immune response.

## Introduction

*Haemophilus influenzae* is an extracellular, gram-negative pathogen that is a primary causative agent of otitis media in children and is also frequently isolated from adults with pneumonia and exacerbations of chronic obstructive pulmonary disease (COPD) [1]–[5]. Colonization of the upper respiratory tract with *H. influenzae* is common and is the first step in disease development, as *H. influenzae* carriage is associated with recurrent otitis media episodes in children [6], [7]. While the Hib conjugate vaccine has greatly reduced the burden of disease caused by type b *H. influenzae* [8], [9], non-typeable *H. influenzae* (NTHi) strains, which are unencapsulated, remain a common source of respiratory tract infections. Vaccine strategies targeting NTHi strains are complicated by the high variability of outer membrane antigens [10], [11]. One of the structurally diverse molecules on the surface of *H. influenzae* is the lipopolysaccharide (LPS).

The LPS of *H. influenzae* is truncated compared to the LPS of other gram-negative bacteria. It contains no repetitive O antigen side chains and is also referred to as lipooligosaccharide (LOS) [12], [13]. *H. influenzae* LPS consists of lipid A attached to 3-deoxy-D-manno-oct-2-ulosonic acid (KDO), with three conserved inner core heptoses to which various oligosaccharide extensions, and other non-carbohydrate molecules, can be attached [14]. Mass spectrometry (MS) analysis of different *H. influenzae* isolates has revealed a significant level of diversity in LPS structures [15]–[21]. For example, the length and composition of the hexose extensions from the inner core heptoses, as well as the attachment of molecules such as sialic acid and glycine, varies both between different strains and within glycoforms of the same isolate. A major source of LPS variability in *H. influenzae* is on-off switching, or phase variation, involving LPS biosynthesis genes [22], [23]. One of the phase variable molecules expressed on *H. influenzae* LPS is phosphorylcholine.

Phosphorylcholine [(CH_3_)_3_N^+^CH_2_CH_2_PO_4_^−^], or ChoP, is a small, zwitterionic molecule that is covalently attached to the LPS through its phosphate group. ChoP is a surface structure of a number of bacteria in addition to *H. influenzae*, particularly those found in the respiratory tract, including *Streptococcus pneumoniae*, *Pseudomonas aeruginosa*, and *Neisseria* species [24]–[26]. ChoP is also a component of eukaryotic membrane lipids in the form of phosphatidylcholine. *H. influenzae* must acquire choline from the environment, and turnover of host lipids can be a major source of choline during colonization [27], [28]. Choline import, phosphorylation, and attachment to *H. influenzae* LPS is controlled by genes in the *lic1* locus. The choline kinase gene *lic1A* contains a tetranucleotide repeat that is responsible for ChoP phase variation. Slipped-strand mispairing within the repeat region of *lic1A* creates a translational on-off switch controlling ChoP expression [29]. As a result, the control of ChoP attachment to the LPS is stochastic, and phase variation occurs at a high frequency [30].

Phase variation of ChoP expression may provide a mechanism for *Haemophilus* to display a variety of phenotypes, allowing rapid adaptation to different host environments. ChoP attachment to the LPS enables recognition by C-reactive protein (CRP), which binds to ChoP and initiates classical pathway complement-mediated killing [31]. In host environments with high levels of CRP, such as in the blood, there is a selective advantage for ChoP− phase variants [32]. In addition, an antibody response can be initiated against LPS epitopes containing ChoP [33], [34]. However, the maintenance of phase variable ChoP expression predicts that there are also advantages for ChoP+ bacteria in select host environments. During *H. influenzae* colonization there is a strong selection for ChoP+ phase variants. This selection has been observed in several animal models of *Haemophilus* colonization, as well as during human carriage [31], [35]–[37]. ChoP expression increases adherence to epithelial cells through interaction with platelet-activating factor receptor (rPAF), which normally binds the ChoP-containing molecule PAF. While *in vitro* experiments have demonstrated the capacity of ChoP+ bacteria to bind rPAF, mice deficient in rPAF have no colonization defect [38], [39]. These data suggest that there are additional host factors involved in the selection for ChoP+ bacteria during colonization. Here, we show that ChoP attachment to the LPS alters the physical properties of the outer membrane and reduces antibody binding to the surface of *H. influenzae*.

## Results

### Adaptive immunity is required for the selection of ChoP+ phase variants during colonization

**Figure 1:**
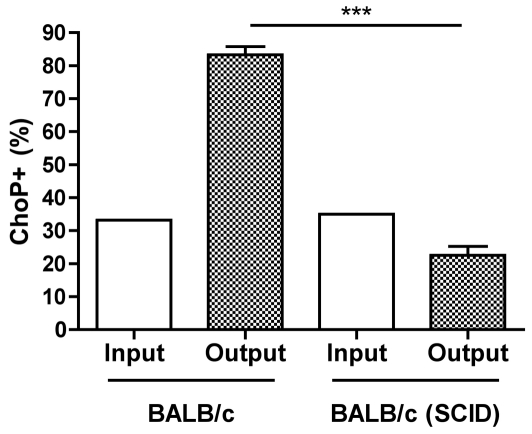
Adaptive immunity is required for selection of ChoP+ phase variants during colonization. BALB/c or BALB/c (severe combined immune deficiency, or SCID) mice were intranasally inoculated with 10^7^ CFU/mL of a 3∶1 mixture of ChoP−∶ChoP+ phase variants of NTHi strain H632. The percentage of ChoP+ phase variants was determined by colony immunoblotting of the inoculum (input, white bars) and the nasal lavage fluid following three days of colonization (output, stippled bars). Values are derived from five mice per group ± SD, ***p<.0001.

The role of adaptive immunity in the selection of ChoP+ phase variants was examined *in vivo* using a murine model of nasopharyngeal colonization. The percentage of ChoP+ phase variants was determined by colony immunoblotting following colonization with a mixture of ChoP+/− variants of strain H632, an NTHi strain that is able to colonize mice [40]. The proportion of ChoP+ phase variants in the output (colonizing) population was substantially greater than that in the input (inoculum) after challenge of immune competent, (wild-type) BALB/c mice (Figure 1). In contrast, there was no evidence for a selection of ChoP+ phase variants following colonization in BALB/c mice lacking an adaptive immune system (severe combined immune deficiency, or SCID) [41]. These results demonstrate that adaptive immunity is important for the selection of ChoP+ phase variants during colonization.

### ChoP expression reduces antibody binding to the surface of *H. influenzae*

**Figure 2:**
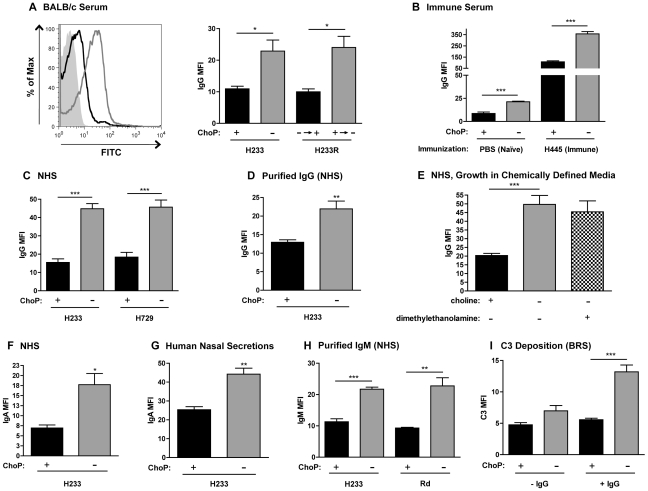
ChoP expression decreases antibody binding to the bacterial surface. Flow cytometric analysis was used to quantify antibody binding to bacterial cells. Representative histogram of BALB/c serum IgG binding to phase variants of NTHi strain H233, and the mean fluorescence intensity (MFI) for binding to H233 phase variants and revertants of the originally selected phase variants (A). MFI of IgG binding from the serum of naïve (intranasally inoculated with PBS) or immune (intranasally inoculated with constitutive ChoP− type b strain H445) BALB/c mice to phase variants of type b (unencapsulated) strain H625 (B). The MFI of normal human serum (NHS) IgG binding to phase variants of NTHi strains H233 and H729 (C) and purified IgG for strain H233 (D). The MFI of NHS IgG binding for Rd constitutive ChoP+ strain H491 grown in CDM alone (grey bar) or supplemented with choline ((CH_3_)_3_N^+^CH_2_CH_2_, black bar) or dimethylethanolamine ((CH_3_)_2_NCH_2_CH_2_, stippled bar), (E). The MFI of IgA binding for phase variants of H233 using NHS (F) or pooled human nasal secretion IgA binding (G). The MFI of IgM purified from NHS binding for phase variants of H233 and Rd (H). The MFI of C3 binding from BRS to phase variants of H233 in the presence or absence of purified IgG (from NHS) (I). ChoP+ variants are shown in black bars, ChoP− variants are shown in grey bars. Values are derived from at least three independent experiments ± SD, *p<.05, **p<.01, ***p<.001.

The importance of natural, or pre-existing, antibody in limiting *H. influenzae* colonization has been demonstrated previously [40]. These data led to an examination of the impact of ChoP expression on antibody binding by flow cytometry. It was found that ChoP+ variants had reduced antibody binding to their surface compared to ChoP− variants (Figure 2). The ChoP+ phase variant of NTHi strain H233 had decreased binding of natural IgG using both serum from (wild-type) BALB/c mice (Figure 2A) and normal human serum (NHS; Figure 2C). Revertants of the originally selected phase variants of strain H233, as well as ChoP+/− phase variants in another NTHi strain, H729, showed a similar effect (Figure 2A, C). The ChoP-mediated reduction of the binding of natural antibody in serum (naïve control) was also observed in the serum of mice previously intranasally immunized with *H. influenzae* (Figure 2B). To confirm the difference in antibody binding was not dependent on other serum components, the effect of ChoP on binding of IgG purified from NHS was also examined, with the same result (Figure 2D). This binding assay was also conducted using a mutant strain of Rd with constitutive ChoP+ expression (phase locked ChoP+), H491. This strain was grown in chemically defined medium (CDM) with or without choline. The binding of IgG in NHS to H491 was reduced when choline was added to the CDM, compared to CDM without choline (Figure 2E). ChoP expression also reduced binding of IgA from NHS (Figure 2F), from human nasal secretions (Figure 2G), and IgM purified from NHS (Figure 2H).

To examine whether there was an additional effect of ChoP expression on binding of complement component C3, baby rabbit serum (BRS) was used as a source of complement without natural antibody to *H. influenzae*. While there was no difference C3 binding to ChoP+/− phase variants in BRS alone, the ChoP+ phase variant of strain H233 had reduced C3 binding in the presence of BRS with purified IgG from NHS (Figure 2I). Collectively, these data demonstrate that ChoP expression results in decreased antibody binding, which limits complement deposition, on the surface of *H. influenzae*.

### ChoP expression reduces binding of LPS-specific, bactericidal antibody

**Figure 3:**
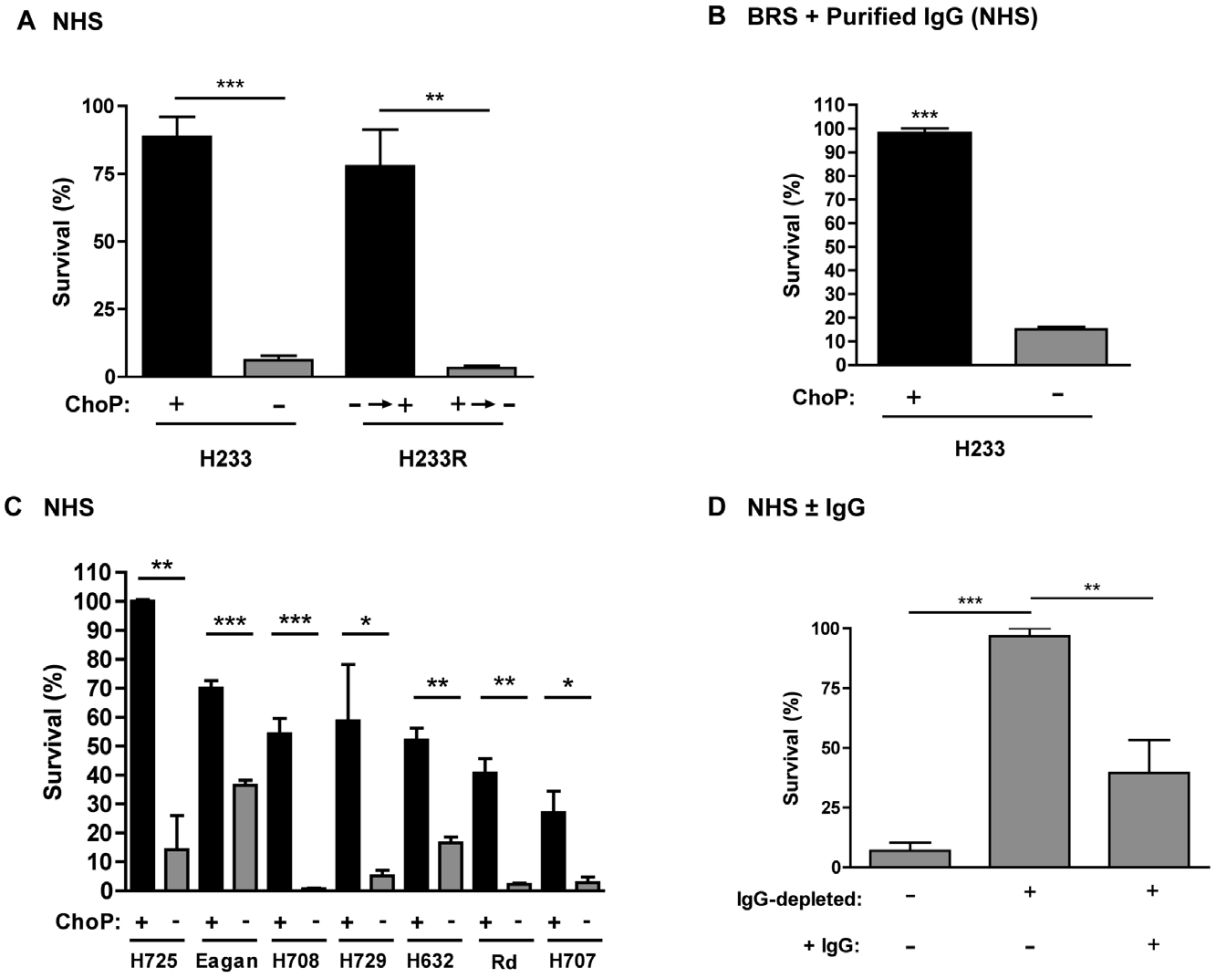
ChoP expression protects against bactericidal antibody. Percent survival in C-reactive protein (CRP)-depleted NHS (relative to complement-inactivated control) is shown for phase variants of NTHi strain H233 and revertants of the originally selected phase variants (A). Percent survival in purified IgG from NHS with BRS (complement source) for phase variants of H233 (B). Percent survival in CRP-depleted NHS for constitutive ChoP+ and ChoP− mutants in Rd, phase variants of type b strain Eagan, and multiple NTHi strains (C). Percent survival of NTHi strain H233 ChoP− phase variant in NHS, IgG-depleted NHS, or in IgG-depleted NHS supplemented with purified IgG, is shown as indicated (D). ChoP+ variants are shown in black bars, ChoP− variants are shown in grey bars. Values are derived from at least three independent experiments ± SD, *p<.05, **p<.01, ***p<.001.

The classical pathway of complement-mediated killing can be initiated by binding of CRP or bactericidal antibody to *H. influenzae*. In order to determine if ChoP expression affects classical pathway complement-mediated killing following antibody binding, NHS depleted of C-reactive protein (CRP) was used as an antibody and complement source for bactericidal assays. ChoP+ phase variants had increased survival in CRP-depleted NHS compared to ChoP− variants of strain H233, as well as revertants of the originally selected phase variants (Figure 3A). There was also increased survival of the ChoP+ phase variant of H233 using IgG purified from NHS (in the same concentration as that used for flow cytometry in Figure 2D) with BRS as a complement source (Figure 3B). The increased survival of ChoP+ bacteria in the presence of CRP-depleted NHS was observed for multiple other NTHi strains, constitutive ChoP+ and ChoP− mutants in Rd, and an *H. influenzae* type b strain (Eagan) (Figure 3C). Serum was IgG-depleted to determine whether the difference in survival of ChoP variants was dependent on antibody. In IgG-depleted NHS there was a recovery of survival for the ChoP− variant of H233, while the addition of purified IgG restored killing (Figure 3D). No differences in survival were detected for bactericidal assays conducted in BRS as a source of complement without antibody, or in NHS with MgEGTA buffer, which allows alternative pathway complement-mediated killing only (not shown). Collectively, these results suggest that ChoP expression increases survival in the presence of bactericidal antibody.

**Figure 4:**
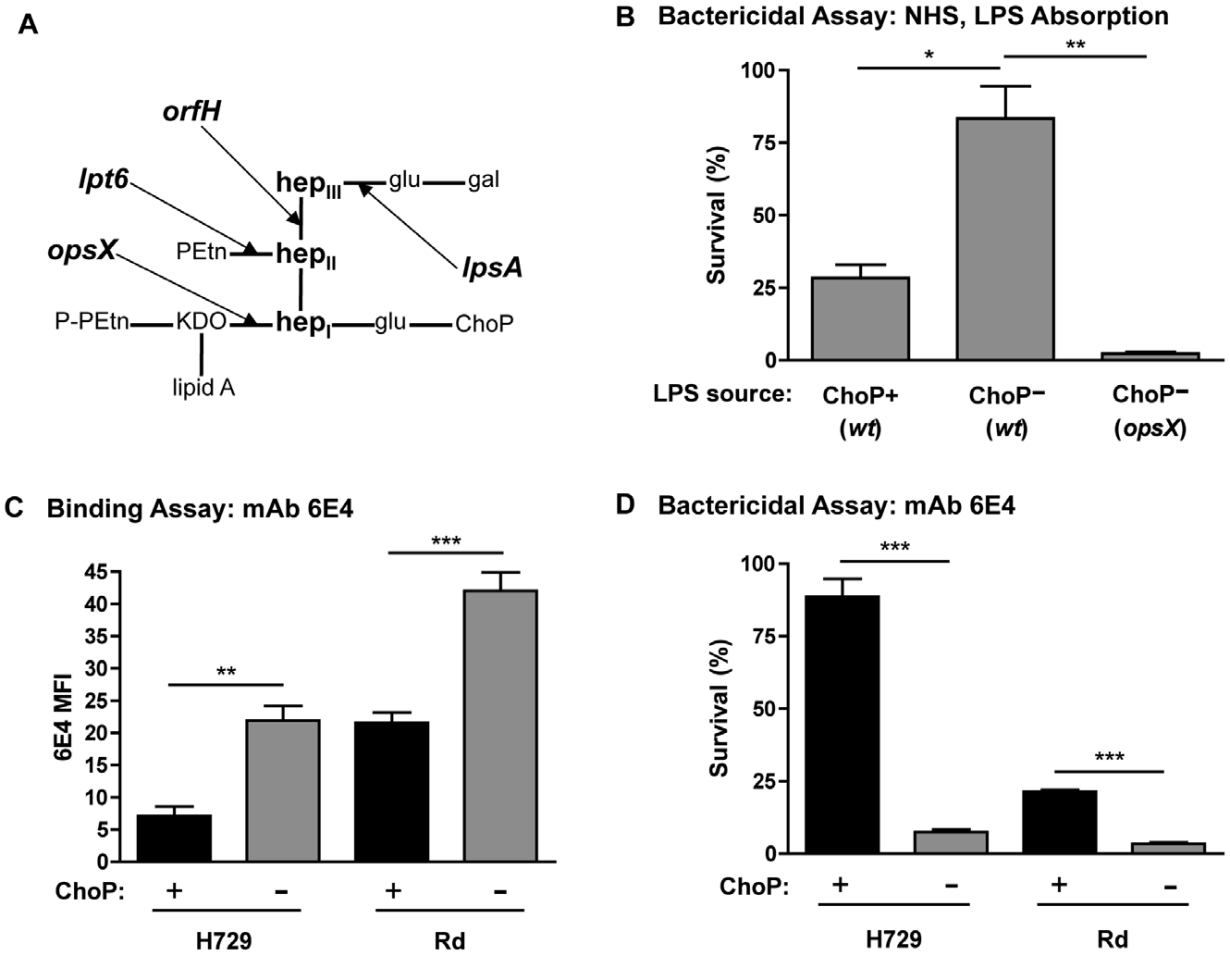
ChoP expression reduces binding of LPS bactericidal antibody. Rd LPS structure with arrows indicating sites of truncations for LPS biosynthesis mutants (A). Percent survival (relative to complement-inactivated control) for Rd constitutive ChoP− strain H446 in NHS that was absorbed with: purified Rd LPS from the constitutive ChoP+ mutant, the constitutive ChoP− mutant, or an *opsX* mutant (B). Flow cytometric analysis of the MFI of mAb 6E4 binding to phase variants of NTHi strain H729 and constitutive ChoP+ and ChoP− mutants in Rd (C). Percent survival in BRS (complement source) and mAb 6E4 for phase variants of NTHi strain H729 and constitutive ChoP+ and ChoP− mutants in Rd (D). ChoP+ variants are shown in black bars, ChoP− variants are shown in grey bars. Values are derived from at least three independent experiments ± SD, *p<.05, **p<.01, ***p<.001.

The potential targets on the surface of *H. influenzae* that are protected from antibody binding by ChoP expression were examined using antibody-depleted NHS and mAbs. Purified LPS was used to absorb LPS–specific antibodies from NHS prior to conducting bactericidal assays. LPS antibody pre-absorption with *wt*, constitutive ChoP− LPS resulted in increased survival of the constitutive ChoP− mutant in Rd, H446 (Figure 4B). In contrast, there was no increase in survival of the constitutive ChoP− mutant after pre-absorption with ChoP− LPS from the Rd *opsX* mutant strain, which is highly truncated with no KDO heptose extensions (Figure 4A, B). To confirm that the LPS pre-absorbed serum retained complement activity, purified IgG was added back to the LPS pre-absorbed serum, and killing was observed (not shown). These results indicate that the majority of the bactericidal antibody affected by ChoP expression in NHS is LPS oligosaccharide-specific. Of note, that there was a greater increase in the survival of the Rd constitutive ChoP− mutant H446 following NHS pre-absorption with ChoP− LPS, compared to pre-absorption with ChoP+ LPS, a result that correlated with the effect of ChoP on antibody binding (Figure 4B). While purified LPS may not accurately mimic the environment of the outer membrane, this result demonstrates that the same decrease in antibody binding observed in ChoP+ whole bacteria is also observed for ChoP+ LPS.

The mAb 6E4, which binds *H. influenzae* LPS [42], was used to examine the effect of ChoP on antibody binding to a specific LPS epitope. ChoP expression reduced 6E4 binding and increased survival following incubation in 6E4 with BRS as a complement source (Figure 4C, D). This was observed for NTHi strain H729 and for the constitutive ChoP+ mutant in Rd, H491. These results confirmed that ChoP expression provides protection against bactericidal antibody binding to LPS oligosaccharide epitopes.

### LPS structural requirements for the effect of ChoP on antibody binding

**Figure 5:**
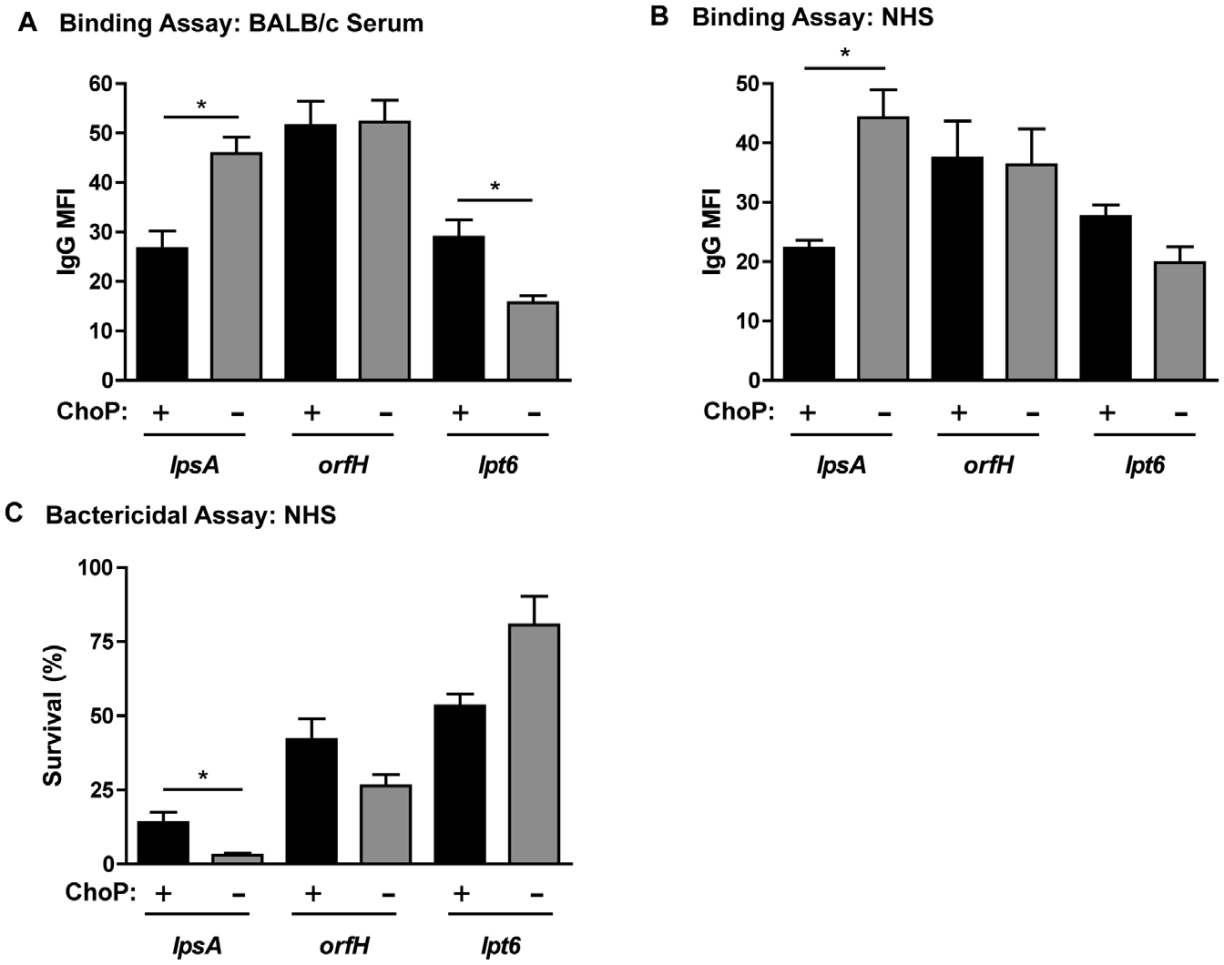
Core LPS structures are required for ChoP-mediated protection against antibody binding. Flow cytometric analysis of the MFI of IgG binding to phase variants of Rd LPS mutant strains *lpsA*, *orfH*, and *lpt6* following incubation in BALB/c serum (A) or NHS (B). Percent survival in CRP-depleted NHS (relative to complement-inactivated control) for phase variants of Rd mutant strains (C). ChoP+ variants are shown in black bars, ChoP− variants are shown in grey bars. Values are derived from at least three independent experiments ± SD, *p<.05, **p<.01.

A selection of LPS mutants in Rd was used to investigate the importance of the LPS molecular environment for ChoP-mediated protection against antibody binding (Figure 4A). The ChoP+ phase variant of the *lpsA* mutant strain, which no longer has Hep_III_ hexose extensions, maintained reduced antibody binding and increased survival in the presence of CRP-depleted NHS (Figure 5). In contrast, there was no longer a protective effect of ChoP expression on antibody binding and complement-mediated killing for the *orfH* and *lpt6* mutant strains. The *orfH* mutation results in a lack of Hep_III_, while the *lpt6* mutation prevents attachment of a conserved phosphoethanolamine molecule to Hep_II_.

**Figure 6:**
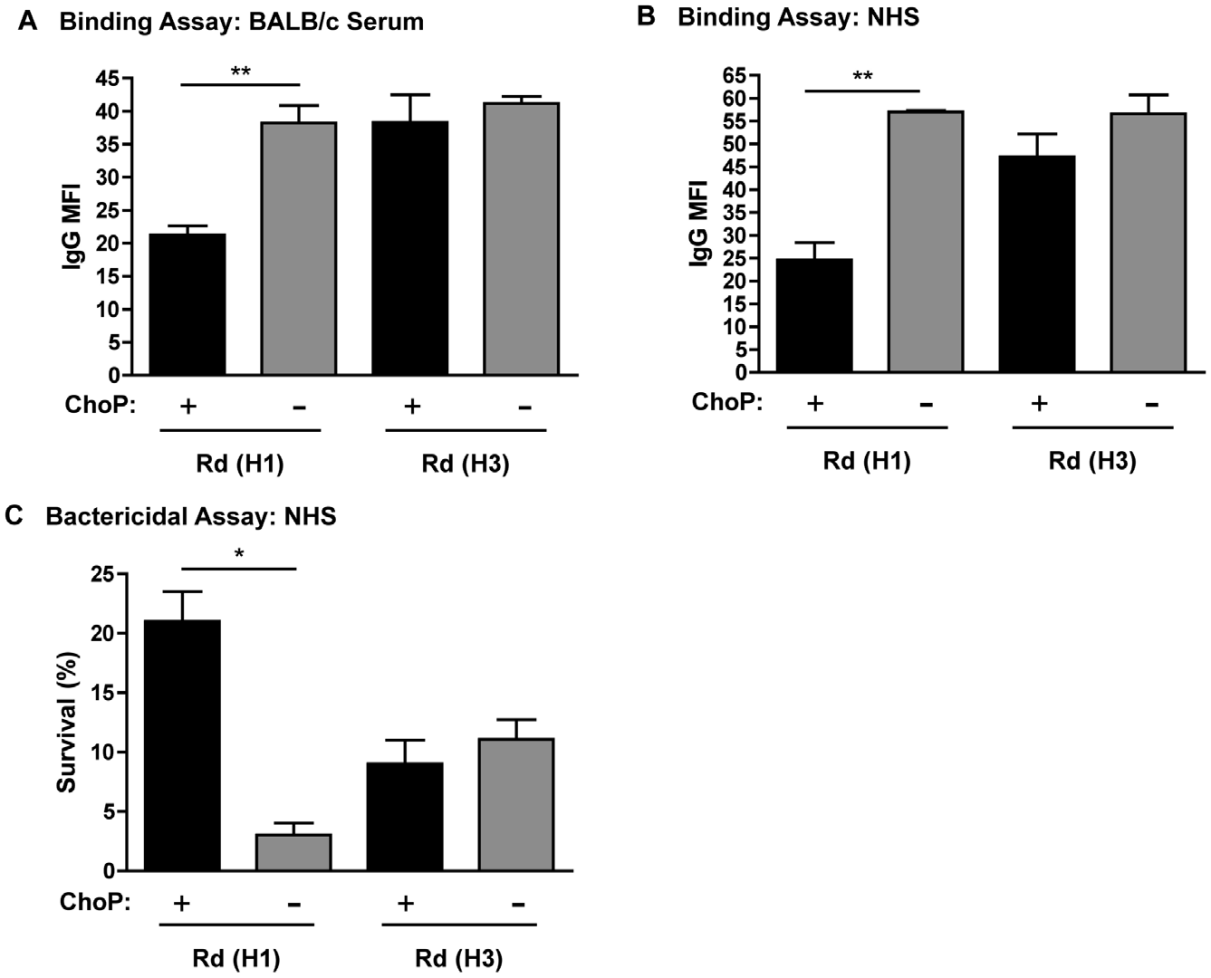
Position of ChoP affects ChoP-mediated protection against antibody binding. Flow cytometric analysis of the MFI of IgG binding to phase variants of Rd *lic1D* exchange mutants; H491 with Rd *lic1D* (Heptose_I_, or H1), and H457 with Eagan *lic1D* (Heptose_III_, or H3), from BALB/c serum (A) or NHS (B). Percent survival (relative to complement-inactivated control) was determined following incubation in CRP-depleted NHS (C). ChoP+ variants are shown in black bars, ChoP− variants are shown in grey bars. Values are derived from at least three independent experiments ± SD, *p<.05, **p<.01.

ChoP can be attached to hexose extensions from multiple heptose residues, most commonly Hep_I_ and Hep_III_ [20], [43], [44]. The importance of the position of ChoP for its effect on antibody binding was examined using *lic1D* exchange mutants, as the *lic1D* allele dictates the position of ChoP attachment [45]. In Rd, ChoP is attached to a hexose extension on Hep_I_ (HI). Alteration of the position of ChoP in Rd from H1 to a hexose extension on Hep_III_ (H3) resulted in the loss of ChoP-mediated protection against antibody binding and antibody-dependent bactericidal activity (Figure 6). Collectively, these data demonstrate that the constituents of the oligosaccharide and the molecular environment of ChoP are both important for the effect of ChoP on antibody binding and resistance to antibody-dependent, complement-mediated killing.

### ChoP expression alters the physical properties of the outer membrane

**Figure 7:**
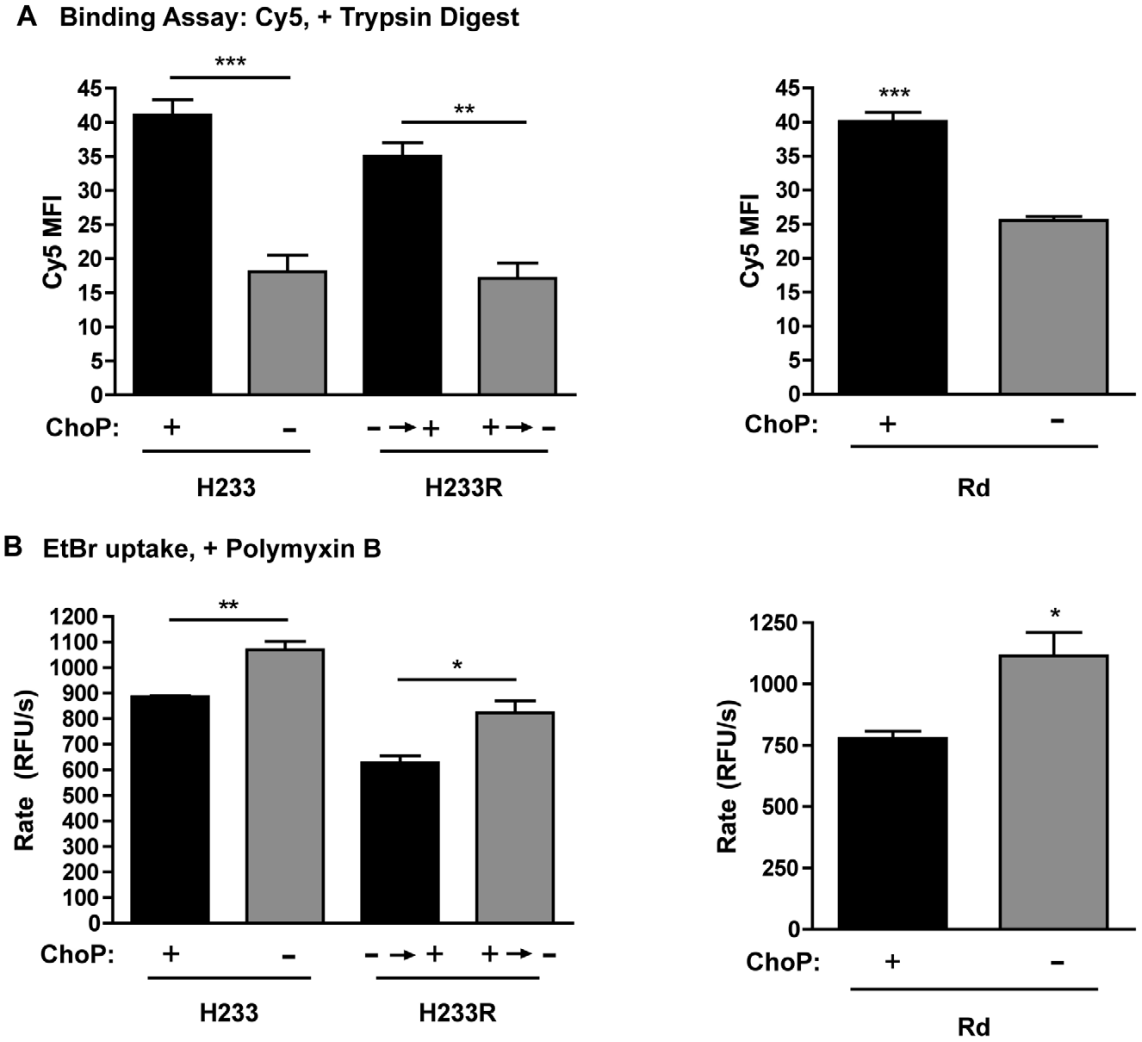
ChoP expression affects outer membrane accessibility and barrier function. Flow cytometric analysis of the MFI of Cy5 binding to outer membrane surface proteins following trypsin digestion for phase variants of NTHi strain H233 and revertants of the originally selected phase variants and constitutive ChoP+ and ChoP− mutants in Rd (A). Rate of ethidium bromide (EtBr) uptake, expressed in relative fluorescent units per second (RFU/s), in the presence of polymyxin B for phase variants of NTHi strain H233 and revertants and constitutive ChoP+ and ChoP− mutants in Rd (B). ChoP+ variants are shown in black bars, ChoP− variants are shown in grey bars. Values are derived from at least three independent experiments ± SD, *p<.05, **p<.01, ***p<.001.

We next investigated the mechanism for ChoP-mediated protection against antibody binding, considering two possibilities; 1) steric hindrance, where ChoP obscures key epitope(s) and 2) ChoP alteration of the physical properties of the outer membrane, resulting in decreased membrane accessibility. In order to test the effect of ChoP expression on the ability of molecules other than antibodies to access the membrane, trypsin sensitivity was compared for ChoP+ and ChoP− phase variants. The fluorescent dye Cy5, which labels lysine residues, was used to quantify the exposure of outer membrane surface proteins by flow cytometry. Following trypsin digestion, there was decreased Cy5 binding to the ChoP− phase variant of strain H233, as well as the ChoP− revertant of the original ChoP+ phase variant (Figure 7A). The same effect was observed for the constitutive ChoP− mutant in Rd, H446 (Figure 7A). The reduction in Cy5 binding following trypsin digestion suggests that ChoP expression affects the general accessibility of molecules, including antibodies, to outer membrane targets.

In order to further examine the effect of ChoP on the physical properties of the outer membrane, membrane barrier function was compared in ChoP+ and ChoP− phase variants. Bacterial uptake of the dye ethidium bromide (EtBr) was used to measure the effect of ChoP on the permeability of the outer membrane. The rate-limiting step for EtBr uptake is transversal of the outer membrane [46]. In the presence of a low concentration of polymyxin B, there was an increased rate of EtBr uptake in the ChoP−, compared to the ChoP+, phase variants of H233 (Figure 7B). The same effect was observed for the revertants of the original variants of strain H233 as well as the constitutive ChoP+ and ChoP− mutants in Rd (Figure 7B). It was necessary to include polymyxin B to cause initial membrane destabilization for dye uptake. While ChoP expression does not affect killing by polymyxin B alone (not shown), the difference in EtBr uptake rates may reflect a difference in outer membrane susceptibility to polymyxin B. The alteration of polymyxin B-induced EtBr uptake demonstrates that ChoP expression strengthens the barrier function of the outer membrane.

Differences in membrane barrier function often correlate with changes in the gel-to-liquid crystalline phase transition temperature, or T*m*, which can be determined by differential scanning calorimetry (DSC) [47]. In order to test the effect of ChoP expression on the T*m* of *H. influenzae* LPS, DSC was performed on LPS purified from ChoP+ and ChoP− bacteria. The T*m* of LPS from the Rd constitutive ChoP+ strain H491 was determined to be 29.8±.2°C, while the T*m* of the constitutive ChoP− mutant strain H446 was significantly higher, at 34.3±.1°C (p<.0001). Phase transition temperatures were independent of Mg^2+^ concentration.

**Figure 8:**
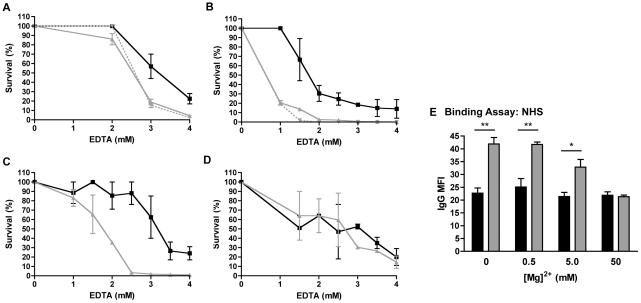
ChoP expression increases outer membrane integrity. Percent survival following incubation in EDTA was determined for ChoP+ (black solid), ChoP− (grey solid), and ChoP−,Galα1-4Gal+ (grey dashed) phase variants of NTHi strain H233 (A). Percent survival in EDTA for Rd constitutive ChoP+ strain H491 grown in CDM alone (grey solid), or supplemented with choline (black solid) or with dimethylethanolamine (grey dashed) (B). Percent survival in EDTA for Rd *lic1D* exchange mutants, Rd *lic1D* (H1) (C) and Eagan *lic1D* (H3) (D). Flow cytometric analysis of the MFI of NHS IgG binding in the presence of 0–50 mM Mg^2+^ for ChoP+ (black bars) and ChoP− (grey bars) phase variants of NTHi strain H233 (E). Values are representative of three independent experiments performed in duplicate ± SD (A–D), or are derived from three independent experiments ± SD *p<.05, **p<.01 (E).

The effect of ChoP on the integrity of the outer membrane was examined by comparing EDTA sensitivity in ChoP+ and ChoP− phase variants. EDTA treatment chelates the divalent cations that are important for maintaining LPS interactions and membrane stability [48]. The ChoP+ variant of strain H233 had increased resistance to EDTA, compared to the ChoP− variant (Figure 8A). ChoP revertants of these variants showed the same trend (not shown). Growth of the Rd constitutive ChoP+ strain H491 in CDM with choline also resulted in increased EDTA resistance, compared to survival following growth in CDM without choline (Figure 8B). In contrast, the expression of a digalactoside residue (Galα1-4Gal) in strain H233, detected by the mAb 4C4, had no impact on EDTA resistance (Figure 8A).

The importance of the position of ChoP for its effect on outer membrane integrity was also investigated. For the Rd *lic1D* exchange mutant strains, only ChoP in the H1, but not H3, position resulted in increased EDTA resistance (Figure 8C, D). These results correlate with the effect of ChoP position on antibody binding, suggesting that the same structural requirements for ChoP-mediated reduction of antibody binding are necessary for its effect on the integrity of the outer membrane. Next, the effect of divalent cation concentration on antibody binding was explored. Increasing the Mg^2+^ concentration (up to 50 mM) resulted in reduced antibody binding to the ChoP− phase variant of H233 (Figure 8E). These results demonstrate that excess Mg^2+^, which increases the stability of the outer membrane [49]–[51], can correct for the difference in antibody binding between ChoP+ and ChoP− variants. Together, these data indicate that ChoP expression alters the physical properties of the outer membrane, and that these effects correlate with the reduction of antibody binding in ChoP+ phase variants.

### ChoP structural requirements for its effect on antibody binding and outer membrane integrity

ChoP is a zwitterionic molecule with a positively charged quaternary amine group, which may be important for its effect on antibody binding and the physical properties of the outer membrane. The structural components of ChoP that are required for its effect on antibody binding and the integrity of the outer membrane were examined using a strain that incorporated a ChoP analog, dimethylethanolamine phosphate [(CH_3_)_2_NCH_2_CH_2_PO_4_^−^]. This ChoP analog differs from ChoP by a single methyl group, which reduces the positive charge on the amine group with minimal alteration of the overall structure. Incorporation of the ChoP analog into the LPS of the Rd constitutive ChoP+ strain H491 grown in CDM+dimethylethanolamine was confirmed by MS (not shown). H491 grown in CDM+dimethylethanolamine had a similar level of antibody binding as when the strain was grown in CDM alone (Figure 2E). In addition, while H491 grown in CDM+choline had increased EDTA resistance compared to when it was grown in CDM alone, H491 grown in CDM+dimethylethanolamine showed the same percent survival in EDTA as when it was grown in CDM alone (Figure 8B). While it cannot be ruled out that the single methyl group impacts the steric inhibition of antibody binding, these results suggest that the positively charged quaternary amine on ChoP is important for the reduced antibody binding and increased outer membrane integrity observed in ChoP+ phase variants.

## Discussion

Understanding the requirements for *H. influenzae* colonization is integral to the effort to reduce the burden of NTHi-associated disease. *H. influenzae* is susceptible to antibody-dependent, classical pathway complement-mediated killing *in vitro*, and this may be an important mechanism for host control of *H. influenzae in vivo*. For example, human patients with primary antibody deficiencies have persistent colonization and higher rates of disease from NTHi strains [52]. In this light, bacterial factors that affect antibody recognition could play a major role in *H. influenzae* survival during colonization. In this study, it was found that ChoP+ phase variants have reduced binding of antibody, including antibody binding to LPS epitopes, as well as increased survival in the presence of antibody-dependent, complement-mediated killing. While there was no difference in C3 deposition or bacterial survival for ChoP+/− phase variants in the absence of antibody, the experiments conducted in the present study do no exclude the possibility that ChoP expression affects binding of other classical pathway complement components. The major bactericidal antibody from the serum sources in this study was IgG, which can reach the site of colonization through transcytosis [53], [54]. Indeed, nasal lavage fluid from BALB/c mice contains IgG that binds *H. influenzae* targets, including LPS, and a role for complement in limiting *H. influenzae* colonization has also been demonstrated [40]. Increased resistance to bactericidal antibody was observed for ChoP+ phase variants of multiple *H. influenzae* strains, despite the heterogeneity observed in this bacteria [55]. These data demonstrate a novel mechanism for evasion of antibody recognition by *H. influenzae* during colonization. It was also shown that adaptive immunity is required for the increase in the selection of ChoP+ phase variants during colonization. While the short-term colonization model used for the *in vivo* experiments demonstrates a role for ChoP expression in protection against natural antibodies, it was also shown that ChoP+ phase variants have reduced binding of antibody from pre-exposed, immune hosts. Taken together, these results suggest that ChoP expression provides a selective advantage at the mucosal surface during colonization, as ChoP+ bacteria are better protected against antibody binding and antibody-dependent clearance.

ChoP is one of several LPS structural determinants whose attachment to the LPS is controlled by stochastic phase variation [29], [56]. Other phase variable decorations to the LPS have been shown to have an effect on serum resistance. For example, loss of O-acetylation, sialylation, or the digalactoside residue Galα1,4Gal results in increased serum sensitivity, attributed to different mechanisms [57]–[59]. The LPS of *H. influenzae* is highly heterogeneous [60], and phase variation of LPS epitopes may allow bacteria to quickly adapt to the repertoire of antibodies present in different host environments. While it was shown in the current study that ChoP+ phase variants have reduced binding of the mAb 6E4, the full scope of the LPS, or non-LPS, epitopes protected from antibody recognition by ChoP expression remains unknown. As mentioned previously, ChoP attachment to surface structures has been observed in several bacterial species. In addition to the LPS, ChoP has been found in bacteria on teichoic acid, pili, and an elongation factor protein [61]–[63]. It has also recently been shown that an effector protein injected by *Legionella pneumophila* modifies host regulatory factors with ChoP [64]. The current study supplies another example of how the attachment of this ubiquitous molecule modulates the properties of its target.

The importance of the molecular environment of the LPS for ChoP-mediated protection against antibody binding was examined using a set of LPS mutants. It was found that two conserved inner core LPS structures (PEtn and Hep_III_) are required for the reduction of antibody binding in ChoP+ phase variants. This result could be due to the direct loss of epitopes that are normally protected against antibody binding by ChoP expression, or through an indirect effect of these structures on binding of antibody to other LPS epitopes. Previously, it was shown that changing the location of ChoP attachment affects sensitivity to CRP [45]. In accordance with this data, we determined that the position of ChoP attachment to the LPS is important for the effect of ChoP on antibody binding in Rd. While the inner core structure of LPS is conserved among *H. influenzae* strains, ChoP can be attached to hexose extensions off of any of the three inner core heptoses or to a fourth heptose present in some NTHi strains [17], [18], [20]. There are also NTHi strains with a partial duplication of the *lic* locus, resulting in the attachment of two ChoP residues to the LPS in ChoP+ phase variants [65]. Each *H. influenzae* strain may have optimized its LPS structural arrangement to enable ChoP-mediated protection against antibody binding. The variable position of ChoP also argues against its main function being sterically hindering antibody binding to other LPS epitopes.

The finding that ChoP+ phase variants of *H. influenzae* are also less sensitive to trypsin digestion of outer membrane proteins led to the investigation of the effect of ChoP on the physical properties of the outer membrane. The reduced access of a non-antibody molecule to the membrane suggests that the effect of ChoP on antibody binding is due to a general effect on the outer membrane, rather than direct steric hindrance. This concept is supported by a recent study from this lab demonstrating that mutations in *H. influenzae* that change the distribution of phospholipids in the outer membrane result in decreased outer membrane stability and increased antibody binding to LPS epitopes [66].

In support of the hypothesis that ChoP expression affects the physical properties of the outer membrane, it was shown that ChoP+ phase variants have reduced sensitization to treatments that may compromise the outer membrane, such as polymyxin B-induced EtBr permeability. Polymyxin B is a cationic peptide that targets negatively charged residues in the LPS [67]. A previous study demonstrated that ChoP expression reduces sensitivity to killing by the cationic antimicrobial peptide LL-37 [68], further supporting a ChoP-mediated impact on the integrity of the outer membrane. While studies in several bacterial species have shown that lipid A modifications can affect antimicrobial resistance and membrane permeability [46], [49], [69], [70], the data presented in the current paper demonstrate that modifications outside of the lipid A-KDO inner core region can also impact membrane integrity and barrier function. DSC has been used to determine the phase transition temperatures of phospholipid membrane systems as well as purified LPS from various bacterial strains [71], [72]. In the current study, the phase transition temperature for purified LPS from a constitutive ChoP+ strain was found to be reduced compared to that for ChoP− LPS. A similar trend is observed in phospholipid membrane systems, where the addition of lipids containing ChoP reduce membrane permeability and the T*m* [47]. In DSC experiments using LPS isolated from *Salmonella minnesota*, it was found that mutants with reduced oligosaccharide extensions had a lower T*m*, demonstrating that changes to the oligosaccharide, in addition to lipid A alterations, can affect the phase transition temperature [51], [73].

It was shown in the current study that ChoP+ phase variants of *H. influenzae* have increased resistance to membrane disruption by EDTA. EDTA chelates the divalent cations that are important for the stabilization of the outer membrane through association with multiple negatively charged phosphate groups on the LPS [50], [74]. It has been shown that EDTA treatment causes loss of outer membrane organization and shedding of LPS molecules, resulting in reduced membrane integrity [74], [75]. The modification of outer membrane integrity correlated with an effect on antibody binding, as the addition of Mg^2+^ alone resulted in decreased antibody binding. While ChoP is a relatively small structural addition to *H. influenzae* LPS, the presence of the positively charged quaternary amine group may impact the outer membrane by altering charge interactions. It was shown in this study that bacteria that incorporated dimethylethanolamine phosphate instead of ChoP did not have reduced antibody binding or increased EDTA resistance. These data indicate that the positively charged amine group, rather than the negatively charged phosphate group, is required for the effect of ChoP on outer membrane integrity and antibody binding. The expression of molecules with amine groups on lipid A, as well as KDO, has been shown to increase outer membrane stability in other bacteria [76], [77]. Our study suggests a similar effect through modification of the outer core of the oligosaccharide with ChoP. This also supports the notion that a large part of the effect of ChoP on antibody binding is indirect, through alteration of outer membrane accessibility, rather than direct steric inhibition.

In summary, these data indicate that ChoP expression increases the barrier function and integrity of the outer membrane, and these alterations correlate with a reduction in the accessibility and binding of antibody to ChoP+ phase variants. Phase variation of ChoP may be an important consideration in the design of NTHi vaccines targeting LPS epitopes, as selection for ChoP+ phase variants could abrogate vaccine effectiveness. In contrast, vaccines targeting ChoP would select for ChoP− phase variants, and thereby increase effective immune responses to other cell surface epitopes. While phase variation of LPS structures can directly alter the presence of specific epitope(s), these data suggest a novel mechanism whereby ChoP expression affects the ability of antibody to recognize bacterial targets by altering access to outer membrane antigens.

## Materials and Methods

### Ethics statement

This study was conducted according to the guidelines outlined by National Science Foundation Animal Welfare Requirements and the Public Health Service Policy on the Humane Care and Use of Laboratory Animals. The protocol was approved by the Institutional Animal Care and Use Committee, University of Pennsylvania Animal Welfare Assurance Number A3079-01.

### Bacterial strains

All strains are listed in Table 1. Strains were grown in brain heart infusion media (Becton Dickinson Biosciences, Franklin Lake, NJ) supplemented with Fildes enrichment (Remel, Lenexa, KS) and 20 µg/mL β-Nicotinamide adenine dinucleotide hydrate (Sigma, St. Louis, MO), referred to as sBHI. When specified, strains were grown in CDM, prepared as previously described [78]. CDM was supplemented with 300 µM of choline chloride or the choline analog *N,N*-Dimethylethanolamine (Sigma) where indicated. Selection of ChoP and Galα1-4Gal containing phase variants was performed by colony immunoblotting as previously described [79]. Revertants of ChoP variants were selected in the same manner. Each ChoP phase variant population was determined to be over 98% ChoP+ or ChoP− by colony immunoblotting, and constitutive ChoP mutants were 100% ChoP+ (H491) or ChoP− (H446). The percentage of ChoP+ and ChoP− bacteria in each phase variant population remained constant during growth to log phase, as there is no selective pressure on ChoP expression *in vitro*. ChoP+ colonies transferred to a nitrocellulose membrane were detected using a 1∶10,000 dilution of the monoclonal antibody TEPC-15 (Sigma) followed by a 1∶10,000 dilution of alkaline phosphatase-conjugated anti-mouse IgA (Sigma). Colonies expressing the Galα1-4Gal structure were selected using a 1∶10,000 dilution of the monoclonal antibody 4C4 [80] followed by a 1∶10,000 dilution of alkaline phosphatase-conjugated anti-mouse IgG (Sigma).

**Table 1:** List of strains used in this study.

Strain	Description	Reference
H233	NTHi chronic bronchitis clinical isolate A860516	[87]
H632	NTHi otitis media clinical isolate SR7332.P1, Sm^a^	[88]
H707	NTHi COPD^b^ (non-exacerbation) clinical isolate	[66]
H708	NTHi COPD (non-exacerbation) clinical isolate	[66]
H725	NTHi COPD (exacerbation) clinical isolate	[66]
H729	NTHi COPD (exacerbation) clinical isolate	[66]
Rd	Type d, unencapsulated isolate	[89]
H446	Rd with *lic1D*::Km^c^, constitutive ChoP−	[45]
H457 (H3)^d^	Rd with *lic1D* (Eagan)	[45]
H491 (H1)^e^	Rd with *lic1A*Δ(CAAT)*_n_*, constitutive ChoP+	[45]
*lpsA*	Rd *lpsA*::Km	[90]
*lpt6*	Rd *lpt6*::Km	[91]
*opsX*	Rd *opsX*::Km	[90]
*orfH*	Rd *orfH*::Km	[90]
Eagan	Type b clinical isolate	[92]
H625	Eagan, unencapsulated isolate^f^	[93]
H445	Eagan with *lic1D*:Km, constitutive ChoP−	[45]

a Sm, spontaneous streptomycin resistant mutant,

b COPD, chronic obstructive pulmonary disease,

c Km, contains kanamycin resistance cassette,

d H3, ChoP attached to hexose extension from Heptose_III_,

e H1, ChoP in natural position (for Rd) attached to hexose extension from Heptose_I_,

f type b- spontaneous mutant lacking both copies of the *cap* locus.

### Mouse nasopharyngeal colonization

Colonization studies were conducted as described previously [40]. Briefly, mice were intranasally inoculated with 10^7^ CFU/mL of bacteria that were first washed and diluted in phosphate-buffered saline (PBS). ChoP variants were grown separately, followed by combination at a 3∶1 ratio of ChoP− ∶ ChoP+ bacteria by volume prior to inoculation. Nasal lavage fluid was collected in 200 µl of PBS and plated onto sBHI containing 50 µg/mL streptomycin following three days of colonization. Bacterial counts obtained by nasal lavage were comparable to those collected by plating nasopharyngeal tissue homogenates. The percentage of ChoP+ colonies was determined through detection of ChoP by colony immunoblotting.

### Flow cytometric analysis

Antibody binding was detected by flow cytometry as previously described [66]. Briefly, 200 µl reactions containing mid-logarithmic phase bacterial cells in Hank's buffer without Ca2+ or Mg2+ (Gibco, San Diego, CA) supplemented with 5% fetal calf serum (HyClone, Logan, UT) were incubated with primary antibody for 60 min at 37°C. Primary antibody sources included naïve BALB/c serum (1∶200 dilution), NHS (1∶200 dilution), IgG purified from NHS (Sigma, 4.8 µg), IgM purified from NHS (Sigma, 3.7 µg), normal human nasal secretions (1∶200 dilution), mAb 6E4 (1∶100 dilution for H729, 1∶500 dilution for Rd), and BRS (1∶50 dilution). Serum collected from BALB/c mice that had been intranasally inoculated at day 0, 7, and 14 with either PBS (naïve) or 10^7^ CFU/mL of constitutive ChoP− type b strain H445 (immune) was also used as a source of primary antibody (1∶20 dilution). Reactions mixtures were then washed and re-suspended in 1∶200 dilutions of secondary antibody, followed by incubation at 4°C for 60 min. Secondary antibodies included goat anti-mouse IgG-FITC, goat anti-human IgG-FITC, goat anti-human IgA-FITC, goat anti-human IgM-FITC (Sigma), and goat anti-rabbit polyclonal C3-FITC (MP Biomedical Chappel, Irvine, CA). Reaction mixtures were then washed and re-suspended in PBS with 1% bovine serum albumin and 0.5% paraformaldehyde prior to flow cytometric analysis on a BD FACS Calibur flow cytometer (Becton Dickinson Biosciences). A total of 50,000 cells were collected from each reaction mixture, and the MFI of antibody binding was determined using FlowJo software (Tree Star, Ashland, OR).

### Bactericidal assays

Assays were conducted in 200 µl reaction mixtures containing 20 µl of mid-logarithmic phase bacterial cells (OD_620_ 0.5) diluted to 10^5^ CFU/mL in Hank's buffer with Ca^2+^ and Mg^2+^ (Gibco). Following the addition of serum, reaction mixtures were incubated for 45 min at 37°C. One serum source was CRP-depleted NHS, used at a 1∶10 dilution for H233 and Eagan, a 1∶5–1∶10 dilution for other NTHi strains, and a 1∶50 dilution for Rd. CRP was depleted from NHS using immobilized *p*-aminophenyl phosphoryl choline gel, which also results in depletion of anti-ChoP antibodies, according to the manufacturer's protocol (Thermo Scientific, Rockford, IL). Another serum source was a 1∶20 dilution of BRS, to which purified IgG from NHS was added (Sigma, 4.8 µg). IgG depletion of NHS was performed using a Protein G column (GE Healthcare Bio-Sciences, Uppsala, Sweden). IgG eluted from the Protein G column according to manufacturer's instructions was also used as a source of purified IgG (0.25 µg/mL) for bactericidal assays. Survival due to alternative pathway mediated killing alone was determined by comparing survival in BRS alone (no *H. influenzae* antibodies) and by chelating NHS with gelatin veronal buffer containing MgEGTA (Boston Bioproducts, Worcester, MA) [81]. Serum pre-absorption of anti-LPS antibodies was conducted by incubation of NHS (1∶50 dilution) with 1 µg LPS overnight at 4°C. Baby rabbit serum was used as a source of complement without antibody at a 1∶20 dilution for H233, a 1∶10 dilution for H729, and a 1∶25 dilution for Rd. Bactericidal assays with baby rabbit serum were supplemented with IgG purified from NHS (4.8 µg) for H233, or mAb 6E4 at a 1∶10 dilution for H729 and a 1∶100 dilution for Rd. Percent survival was determined relative to complement-inactivated serum, which was incubated for 30 min at 56°C prior to use. Assays to determine EDTA sensitivity were performed by addition of EDTA (1–4 mM) to bacterial cells in sBHI diluted in Hank's buffer with Ca^2+^ and Mg^2+^, followed by incubation for four hours at 37°C. Percent survival was determined relative to no-EDTA controls.

### LPS extraction

LPS extractions were performed using the phenol-chloroform-petroleum ether method as previously described [82], with modifications [83]. Briefly, bacterial pellets were washed sequentially in ethanol, acetone, and petroleum ether prior to lyophilization. The lyophilized samples were re-suspended and mixed overnight in a 2∶5∶8 extraction mixture of phenol∶ chloroform∶ petroleum ether. Following filtration and evaporation of chloroform and petroleum ether, LPS was precipitated from phenol in 5∶1 mixture of acetone∶ diethyl ether. Ultracentrifugation was used to further purify LPS re-suspended in water, followed by lyophilization.

### Trypsin digestion and Cy5 detection of surface proteins

Partial digestion of outer membrane proteins with trypsin was performed as previously described [84]. Briefly, 200 µl of mid-logarithmic phase bacterial cells (OD_620_ 0.5) were washed and re-suspended in 10 mM Tris-HCL, pH 7.5. Following addition of 1 mg/mL trypsin, bacteria were incubated for 2 hrs at 37°C. The cells were then washed and re-suspended in 10 mM carbonate buffer prior to staining with Cy5 according to manufacturer's instructions (GE Biosciences Amersham, Buckinghamshire, UK). Surface proteins with exposed lysines were labeled with 10 µl of Cy5 (40 pmol) in the dark for 20 min, and reactions were stopped with 20 µl of 10 mM lysine. Cells were washed with 10 mM carbonate buffer and re-suspended in PBS with 1% bovine serum albumin prior to flow cytometric analysis on a BD FACS Calibur flow cytometer (BD Biosciences). A total of 50,000 cells were collected from each reaction mixture, and the MFI of antibody binding was determined using FlowJo software (Tree Star).

### EtBr uptake assay

Ethidium bromide was used as a measure of outer membrane permeability as previously described [46]. Bacterial cells grown to stationary phase (OD_620_ 0.8) were re-suspended in PBS, with 15 µg/mL polymyxin B and 6 µM EtBr added directly prior to each measurement. Fluorescence was measured at an excitation wavelength of 544 nm, an emission wavelength of 610 nm using FLUOstar OPTIMA (BMG Labtech, Ortenberg, Germany). EtBr uptake was expressed by RFU/s.

### DSC analysis of LPS phase transition temperature

Purified LPS samples for each strain were diluted to 2 mg/mL in PBS, sonicated, and subjected to three cycles of incubation at 56°C for five min, vortexing 1 min, and cooling to 4°C. Where specified, MgCl_2_ was added to the PBS at 1∶1 and 5∶1 [MgCl_2_]∶ [LPS] molar ratios. Following preparation, samples were stored at 4°C for several hours before running on the DSC instrument. Heat capacity profiles were determined at a scan rate of 60°C/hr over a temperature range of 10–60°C in a high-resolution differential scanning calorimeter (MCS, MicroCal, Amherst, MA). Three consecutive heating and cooling scans were measured per sample. PBS buffer-buffer references were subtracted from sample data, and concentration was normalized based on sample concentration. The Microcal ORIGIN software package was used to progress baselines. The gel-to-liquid crystalline phase transition temperature, T*m*, was determined by integration from baseline to calculate the midpoint of the transition.

### MS analysis

Purified LPS samples were subjected to mild acid hydrolysis and electrospray ionization-mass spectrometry (ESI-MS) was performed as previously described [85].

### Statistical analysis

Differences between groups were assessed for statistical significance using an unpaired Student's *t*-test (GraphPad PRISM4, GraphPad Software, La Jolla, CA).

### Gene reference numbers

The reference numbers for genes mentioned in the text include: *opsX* (0261), *lpsA* (0765), *orfH* (0523) and *lpt6* (0258, 0259) from the Rd database for the published genome sequence [86], as well as *lic1A* (950399) and *lic1D* (950403) GeneID from the NCBI GenBank database.
